# Mosquitoes as a feasible sentinel group for anti-malarial resistance surveillance by Next Generation Sequencing of *Plasmodium falciparum*

**DOI:** 10.1186/s12936-019-2946-0

**Published:** 2019-10-17

**Authors:** Rebecca Smith-Aguasca, Himanshu Gupta, Estefania Uberegui, Mara Maquina, Francisco Saute, Krijn P. Paaijmans, Alfredo Mayor, Silvie Huijben

**Affiliations:** 1 ISGlobal, Hospital Clínic - Universitat de Barcelona, 08036 Barcelona, Spain; 2 Center for Evolution and Medicine, School of Life Sciences, Arizona State University, Tempe, AZ 85287-1701 USA; 3 Centro de Investigação em Saúde da Manhiça (CISM), 1929 Manhiça, Mozambique; 4 The Biodesign Center for Immunotherapy, Vaccines and Virotherapy, Arizona State University, Tempe, AZ 85287-1701 USA

**Keywords:** Malaria, Drug resistance, *k13*, *pfcrt*, *pfmdr1*, *pfdhps*, *pfdhfr*, Sequencing, *Anopheles funestus*, Mozambique

## Abstract

**Background:**

*Plasmodium falciparum* drug resistance surveillance is key to successful disease control and eradication. Contemporary methods that only allow determination of prevalence of resistance are expensive, time consuming and require ethical considerations. A newer method involving Next Generation Sequencing (NGS) permits obtaining frequency of resistance while allowing to detect minority variants in mixed infections. Here, NGS was tested for *P. falciparum* resistance marker detection in mosquito samples as a feasible and suitable alternative for molecular resistance surveillance. *Anopheles funestus* were collected in southern Mozambique using CDC light traps and manual collections. DNA was extracted from either whole mosquito, head-thorax and abdomen separately or pools of five mosquitoes. These samples were screened for *P. falciparum* and if positive for *k13*, *pfcrt*, *pfmdr1*, *pfdhps* and *pfdhfr* mutations related to anti-malarial drug resistance with Sanger sequencing and NGS.

**Results:**

Among the 846 samples screened for *P. falciparum*, 122 were positive by 18S ssrDNA qPCR with an infection rate of 23.6%. No mutations were observed for *k13* and *pfcrt*72-76 and almost zero for *pfmdr*86, but quintuple *pfdhfr/pfdhps* mutations were near fixation and about half of the isolates contained the *pfmdr*184F polymorphism. Similar allele frequencies of resistance markers were estimated with NGS in comparison with the prevalence of markers obtained with the gold standard Sanger sequencing.

**Conclusions:**

Pooled deep sequencing of *P. falciparum* isolates extracted from mosquitoes is a promising, efficient and cost-effective method to quantify allele frequencies at population level which allows to detect known and unknown markers of resistance in single and mixed infections in a timelier manner. Using mosquitoes as sentinel group and focusing on allele frequency opposed to prevalence, permits active surveillance across a more homogeneous geographical range.

## Background

Currently, most malaria control programmes include vector control, early diagnosis and effective treatment of clinical cases [1]. However, *Plasmodium falciparum* resistance to anti-malarial drugs is one of the main challenges for malaria control in endemic countries since resistant parasites are widespread and continue to evolve in response to the selective pressure applied [2–5]. Chloroquine (CQ) and sulfadoxine–pyrimethamine (SP) had to be discontinued for clinical malaria treatment following increased morbidity and mortality associated with resistance in the past decades. The same could happen in the near future to artemisinin-based combination therapy (ACT), the current first-line therapy, if an alternative would be available [4–8]. Resistance to CQ and SP arose in South-East (SE) Asia and spread to Africa [9–11]. Similarly, concern about the possible expansion or emergence of resistance to artemisinin or to its partner drug in the ACT in Africa has been raised due to parasites with increased clearance time spreading throughout SE Asia [5, 9, 10, 12, 13]. Due to lack of an alternative anti-malarial drug with the same level of efficacy and tolerability at present and in order to achieve successful disease control and eradication, it is fundamental to understand the prevalence and geographical distribution of drug resistance. This requires (1) having up-to-date information on efficacy of these therapies in different areas, (2) establishing an early intervention system and (3) understanding more about the principles of spread of resistance in different areas [2, 3, 14].

Resistance surveillance may be done in several different ways: in vivo, ex vivo/in vitro and by mapping molecular markers [5, 15]. In vivo studies, such as drug efficacy trials, are the gold standard [7, 16, 17] where resistance is characterized by treatment failure or delayed parasite clearance in patients [4, 16, 18]. They are relatively easy to standardize and do not require complex equipment [5]. However, drug efficacy trials are difficult to carry out due to the need of patient follow up (at least 28 days or 42–63 days for drugs with longer half-lives), financial costs, ethical clearance processes and the added logistical challenges in low transmission settings, such as a small number of people in treatment [3, 5, 17]. In ex vivo/in vitro resistance studies, parasites are extracted from human blood, grown in culture and exposed to drugs [5, 15]. These studies provide remarkable information on the parasite’s susceptibility, which is defined by measuring growth or replication in the presence of different concentrations of these anti-malarial drugs [5, 15, 17]. Their main advantage is that they allow to gather information on drug susceptibility to individual drugs while avoiding the confounding effects of in vivo studies, such as host immunity or pharmacokinetics [15, 17]. However, ex vivo studies often present difficulties in the comparison of data between different laboratories due to variations among protocols and different criteria when accepting or rejecting data, as its interpretation is mainly based on visual inspection of dose–response curves [15].

Finally, studies of molecular markers of resistance in parasites from human blood samples are the most commonly used for resistance surveillance. These methods are reliable, timely, cost-effective, quantitative and scalable [3, 5, 16]. Moreover, they are relatively easy to implement and interpret and provide useful information on the spread of known resistance markers [5, 17]. Although the presence of these resistance markers is linked to an increased treatment failure, extrapolation to in vivo therapeutic [5, 16, 17, 19] and preventive [20] efficacy is still challenging, added to a lack of methodological standardization [17]. In addition, both ex vivo and molecular markers studies require trained personnel and specialized laboratory facilities [5, 17], although these facilities are becoming more common in malaria endemic countries. A limiting step in all of the above mentioned surveillance strategies is that they are dependent on participation and blood sampling of human subjects. Hence the development and execution of such studies may be expensive, time consuming and need to be ethically justified. Consequently, there are typically a relatively small number of well-studied sites in endemic areas due to logistical and financial limitations [4].

However, the parasite’s life cycle involves the successive infection of another host besides humans: female *Anopheline* mosquitoes. The collection of these vectors does not require complex and invasive tools, medical training or ethical considerations and it has been shown that mosquito stage malaria parasites are useful to perform drug-resistance epidemiological studies [21]. Hence, a more cost-effective alternative to genetic screening of parasites in human blood would be to screen these parasites inside their vector to identify and detect the prevalence of resistant mutants in malaria endemic areas by mapping molecular markers of resistance [3, 7, 22].

Molecular genotyping techniques have been shown to be useful in epidemiological monitoring of resistant *P. falciparum* present in the vectors [22, 23]. PCR–RFLP (polymerase chain reaction–restriction fragment length polymorphism), the traditional molecular genotyping technique for monitoring drug resistance, while relatively easy, economic [24] and fast [7] compared to other molecular techniques, does not allow for the discovery of novel genetic polymorphisms since it targets predefined polymorphisms [5, 25]. Furthermore, it has relatively low sensitivity and may lead to results that cannot be directly compared between studies due to different fragment sizing that can be obtained from the same molecular marker [5, 7, 16, 24, 25]. Sanger sequencing, a newer method and the gold standard, facilitates this, but its application to large-scale surveillance is limited by low throughput; its reagents relatively higher costs, which are directly proportional to the number of specimens genotyped; and the inability to detect polymorphisms at minor frequencies especially in high transmission areas [3, 5, 16]. Next Generation Deep Sequencing (NGS) is the latest technique. Although it requires trained staff [17], as all other molecular techniques, it can potentially overcome most drawbacks of other molecular genotyping methods and allow detection of novel mutations and minority variant genotypes in mixed infections and quantification of allele frequencies in mixed genotypes, which are usually classified as mutant and, therefore, avoid neglecting the presence of wild-type parasites [3, 5, 26–28].

Moreover, it permits higher throughput, sensitivity, resolution and scalability by pooling all samples, allowing gathering data on the frequency of resistance alleles in a certain area [16, 17, 28]. It is, therefore, important to consider whether the most relevant resistance data is on the level of prevalence of resistance (number of individuals infected with a parasite containing resistance marker) or overall allele frequency. To understand the evolutionary dynamics, allele frequencies need to be assessed in order to determine how fast an allele is spreading through a population. Standard surveillance techniques do not allow to obtain information on frequency of resistance alleles in the parasite population. However, NGS has been identified as a method to determine resistant allele frequencies in a population [3] and can potentially benefit the identification of circulating drug-resistant alleles of individual parasites before they are even selected by drug pressure [17, 26].

Here, the aim was to test whether NGS for *P. falciparum* resistance marker detection in mosquito samples is feasible and if it is a suitable, economic and high-throughput alternative for molecular resistance surveillance.

## Methods

### Mosquito collection

**Fig. 1:**
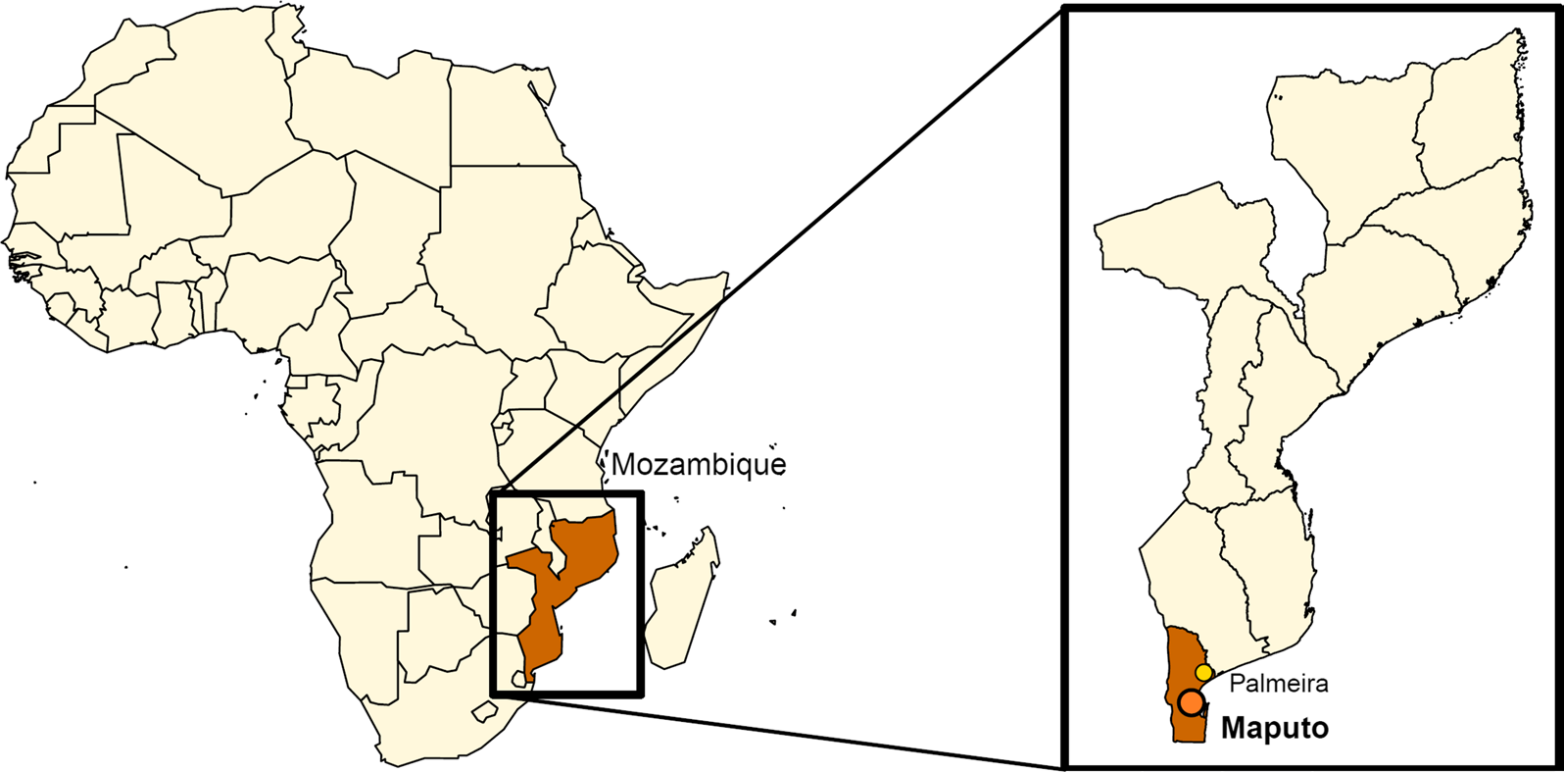
Location of the mosquito collection site

*Anopheles* spp. mosquitoes were collected indoors in thatched and metal roof houses in March 2016 in the town of Palmeira (Manhiça district; S 25°16′40.058″, E 32°52′9.076″), in the South of Mozambique (Fig. 1). It features a tropical savannah climate and exhibits a relatively high malaria transmission (between 100 and 200 cases per 1000 population) [29] although transmission rates have still decreased dramatically in the past decade [30]. Two collection methods were used: early-morning manual collection by mouth aspiration and overnight collection with New Standard Miniature Incandescent Light Trap Model 1012 (John W Hock, USA). In brief, early-morning collections were performed by a manual mouth aspirator whereby female mosquitoes from the *Anopheline* genus were aspirated and transferred to a cup. Miniature light traps were hung next to occupants sleeping under insecticide-treated bed nets (LLINs) and emptied the next morning. Early morning collections and miniature light trap collections were not performed in the same house. Mosquitoes from both collections were subsequently killed in the freezer and stored in tubes with silica gel to desiccate. Afterwards, mosquitoes were morphologically identified to species by trained microscopists using keys of Gillies and Coetzee [31]. The majority of the mosquitoes were identified microscopically as part of the *An. funestus* species complex (99.5%), as found alongside in other studies in the south of Mozambique [32] and considered to be a major human malaria vector in Africa [33]. Therefore, they were the mosquitoes chosen to include in this study, other *Anopheles* species were excluded.

### DNA extraction

DNA extraction from *An. funestus* mosquitoes was carried out using a DNeasy^®^ Blood & Tissue Kit (QIAGEN, Netherlands) following the manufacturer’s instructions for tissue extraction. In brief: mosquitoes were grinded in a lysis buffer and subsequently incubated for a minimum of three hours up to overnight at 56 °C. Lysed samples were subsequently transferred to spin columns and extraction proceeded according to manufacturer’s instructions. Three different DNA extraction methods were compared: (1) DNA was extracted from whole mosquito, (2) mosquitoes were bisected and DNA was extracted from head/thorax (sporozoites only, either in salivary glands and/or circulating in haemolymph) and abdomen (all stages) separately [34–36], and (3) groups of five whole mosquitoes were pooled and grinded into one single sample and DNA was extracted from this mixture.

### Real-time quantitative polymerase chain reaction (qPCR)

Following DNA extraction, real-time quantitative PCR (qPCR) to amplify the 18S small sub-unit rRNA gen (ssrDNA) of *P. falciparum* was carried out on an 7500 Real-Time PCR System (Applied Biosystems, USA) as described in [37] with minor changes. Briefly, a PCR mixture was prepared with 2.5 µl of DNA template, a 5 µM concentration of each primer and a 1.5 µM concentration of TaqMan^®^ TAMRA Probe labelled with 6-carboxy-fluorescein (FAM) as a reporter. A standard curve was prepared using 3D7 samples with a known concentration of parasites and it was run in triplicate with five serially diluted points for each PCR 96 well plate. The results were analysed using default settings on the Applied Biosystems 7500 Fast Real-Time PCR System Sequence Detection Software v1.4.1. *P. falciparum* positive samples were selected for sequencing.

### Mutation frequencies estimation by Sanger sequencing

Five genes were prepared for sequencing to assess mutation frequencies using Sanger and NGS: *P. falciparum k13* propeller gene, chloroquine resistance transporter (*pfcrt)*, multidrug resistance (*pfmdr1*) gene, dihydropteroate synthase (*pfdhps*) and dihydrofolate reductase (*pfdhfr)*.

DNA templates positive by qPCR were amplified on a 2720 Thermal Cycler (Applied Biosystems, USA) following protocols described elsewhere [3, 23, 36, 38, 39]. *K13* propeller gene primers were taken from [36]; *pfdhfr* from [3, 38]; and *pfdhps* from [3] and nested reverse primer for *pfdhps* fragment 1 and nested forward primer for *pfdhps* fragment 2 were newly designed for this purpose with a similar melting temperature (Tm) to those from [3] and GC percentage around 40%. Primers for *pfcrt* were previously described in [39] and for *pfmdr1* in [23] (see Additional file 1: Table S1).

Known positive controls for *k13* propeller gene (k13_1, k13_2, k13_3, k13_4, k13_5 and k13_6) provided by the Institut Pasteur in Cambodia, parasite lines with known *pfcrt* and *pfmdr1* alleles (3D7, 7G8, Dd2 and V1/S) and plasmids with known *pfdhps* and *pfdhfr* alleles (DHPS-V1S, DHPS-PERU, DHPS-MALI, DHPS-DD2, DHFR-V1S, DHFR-FCB, DHFR-50 and DHFR-3D7) were also amplified and sequenced simultaneously with the rest of the samples.

PCR products were run on 2% agarose gels in order to confirm correct amplification. TrackIt™ 100 bp DNA Ladder Invitrogen™ Life Technologies™ was used to size DNA fragments for each amplified gene.

Sanger sequencing of *P. falciparum k13*, *pfcrt*, *pfmdr1*, *pfdhps* and *pfdhfr* genes of those samples with correctly amplified DNA was performed as described elsewhere [23]. *K13* propeller gene primers were taken from [36]; *pfdhfr* from [38]; and *pfdhps* from [3] and reverse primer for *pfdhps* fragment 1 and forward primer for *pfdhps* fragment 2 were newly designed for this purpose in order to meet Genewiz^®^ requirements and with a similar Tm to those from [3]. Primers for *pfcrt* were previously described in [39] and for *pfmdr1* in [23] (see Additional file 1: Table S2). Sequencing was carried out by Genewiz^®^ and results were manually inspected with BioEdit Sequence Alignment Editor v7.0.5 using PlasmoDB reference sequences for all the genes of interest: *k13* (PF3D7_1343700), *pfcrt* (PF3D7_0709000), *pfmdr1* (PF3D7_0523000), *pfdhps* (PF3D7_0810800) and *pfdhfr* (PF3D7_0417200) [40].

### Validation of Next Generation Sequencing (NGS) method

Since amplification intensity appeared similar among samples based on visual inspection of band brightness, 10 µl of each PCR product was taken for pooling. 2% agarose gels were run in order to obtain clear bands which could be purified using QIAquick^®^ Gel Extraction Kit (QIAGEN, Netherlands) following the manufacturer’s instructions. Briefly, samples were incubated at 55 °C for 20 min and then applied to spin columns. Finally, columns were incubated with the elution buffer for 10 min before final elution. This same procedure was followed for 3D7 amplified for all genes as a control.

Qubit quantification of the eluted DNA was performed. Equimolar pooling of gene amplicons to a molarity higher than 4 nM was prepared for each pool: field samples and 3D7 control. In the core genomics facility IDIBAPS (August Pi i Sunyer Biomedical Research Institute), a Nextera DNA library was prepared for both tubes and their size and concentration were checked with 4200 TapeStation Instrument (Aligent, USA) and KAPA Library Quantification Kit Illumina^®^ Platforms (Roche, USA), respectively. After, NGS was performed in duplicate for each of the tubes also by IDIPABS on the MiSeq^®^ System (Illumina, USA) (see Additional file 1: Figure S1).

### Data analysis

Fisher’s exact test was performed to determine correlation between head/thorax and abdomen positivity rate. χ-squared test and Fisher’s exact test were calculated to compare the number of positive mosquitoes for each extraction method (whole body, bisected and pooled) and per collection method (miniature light traps or mouth aspirator). Parasite density (number of parasites per sample) was log-transformed to meet normality assumptions. The geometric mean parasite density and standard deviation (including Taylor’s expansion) were determined. Welch two sample t-test and analysis of variance (one way ANOVA) were conducted to analyse parasite density (number of parasites per sample) for each collection and extraction method, respectively. Statistical analyses were performed using R version 3.3.2 [41]. Twenty-four samples of the manual collected mosquitoes were excluded from infection rate calculations (12 head/thorax, 12 abdomen) due to an error in the extraction procedure, which could have impacted *P. falciparum* detection probability.

Mutation prevalence obtained by Sanger sequencing analysis were calculated for each gene and per position based on the number of samples called by the software as wild-type or mutant alleles. Out of these, mixed alleles were visually observed in the chromatogram. Sequences were also screened for novel mutations.

Each NGS run replicate was analysed independently on Ubuntu, a Linux distribution. First, data quality control was carried out using the program FastQC version 3.3.2 [42]. Since the sequences analysed presented good quality, subsequent sequence alignment was accomplished using the program Bowtie2 version 2.3.4 using default settings and the software Genome Browse Golden Helix v2 was used to view the alignments [43, 44]. Depth of coverage was calculated using the following formula:

$$\text{cov} erage = \frac{number\;of\;aligned\;reads \times length\;of\;reads}{total\;size\;of\;the\;sample\;sequence}$$

Finally, variant calling against *P. falciparum* 3D7 reference sequence from PlasmoDB and quality filtration were performed using GATK 3.8.1 and Picard Tools 4.0.1 (see Additional file 1: Figure S1) [45]. Phred quality score during variant calling was set to 20, to allow for a base call accuracy of 99%, which has been shown to provide accurate results [26, 27, 45–50]. Filtering thresholds were set as recommended by the software developers, which allowed to prioritize sensitivity over specificity [45]. Those variants which passed all the filters were included in further allele frequency analyses. PCR duplicates were not removed as it does not provide downstream added insight in exome sequencing [45]. Allele frequency estimates were calculated by dividing individual unfiltered allele depth by total filtered depth, hence total values do not always add up to 100 [45]. FDR was calculated using Benjamini–Hochberg Procedure. χ-squared test and Fisher’s exact test were calculated to compare the frequency and prevalence of wild-type and mutant alleles for both sequencing techniques.

## Results

### Mosquito infection rate

A total of 995 mosquitoes were collected (429 by mouth aspirator and 566 by miniature light traps). Of those, 846 were screened for *P. falciparum* by qPCR (429 from manual collections and 417 from miniature light traps). Out of these, 122 samples were qPCR-positive, two of which were excluded from the infection rate calculations due to an error in the extraction procedure as precaution, but were included in Sanger and NGS analyses (see Table 1). Infection rate for whole mosquito was 23.6%. Infection rates for head/thorax and abdomen portions separately were lower, 6% and 18.3%, respectively. Moreover, *P. falciparum* detection in a mosquito head/thorax portion was correlated with *P. falciparum* detection in the abdomen of the same specimen (p < 0.001). Positivity rate for pooled samples was 16.7%.

**Table 1:** Number of *P. falciparum* positive mosquitoes (n) per total tested (N) by qPCR captured with miniature light traps and mouth aspirator from each sample type

	CDC light traps n/N (%)	Mouth aspiration (manual) n/N (%)	Total n/N (%)	p-value
Whole body	21/72 (29.2)	13/72 (18.1)	34/144 (23.6)	0.2
Head/thorax	13/150 (8.7)	5/150 (3.3)	18/300 (6)	0.05
Abdomen	36/150 (24.0)	19/150 (12.7)	55/300 (18.3)	0.02
Pooled	11/39 (28.2)	2/39 (5.1)	3/78 (16.7)	0.01
Total	81/411 (19.7)	39/411 (9.5)	120/822 (14.6)	

More samples positive for *P. falciparum* were collected with the miniature light traps than with the manual collection methods (Table 1): head/thorax portions (8.7% vs. 3.3%, p = 0.05), mosquito abdomens (24.0% vs. 12.7%, p = 0.02) and pools of mosquitoes (28.2% vs. 5.1%, p = 0.01). Yet frequency of *P. falciparum* detection in the mosquito as a whole was similar between miniature light trap collected mosquitoes and manual collections (29.2% vs. 18.1%, p = 0.2).

The geometric mean parasite density (number of parasites per sample) was 42.1 with a standard deviation of 76.8. There was no statistically significant difference between the parasite density of mosquitoes in the two collection methods (miniature light traps and mouth aspirator) (t = 0.7, df = 74.1, p = 0.5), and neither was there between extraction methods (whole body, bisection and pooled) (F_3,118_ = 1.5, p = 0.2) (Table 2).

**Table 2:** Geometric mean parasite density (number of parasites per mosquito) and standard deviation of positive mosquitoes tested by qPCR captured with CDC light traps and mouth aspirator from either whole body DNA extraction, head/thorax and abdomen separately or in a pool of five mosquitoes

	CDC light traps		Mouth aspiration (manual)		Total	
	Mean	Standard deviation	Mean	Standard deviation	Mean	Standard deviation
Whole body	40.6	54.3	40.6	87.9	40.6	67.8
Head/thorax	12.3	16.4	57.6	113.2	18.9	30.9
Abdomen	72.3	135.0	29.4	57.7	51.9	100.5
Pooled	59.1	119.5	38.6	0.4	55.3	102.5

### Allele prevalence estimation using Sanger sequencing

122 samples were positive for *P. falciparum*; out of which successfully sequenced sample numbers (forward and/or reverse) per gene were 66 for *k13* (54.1%), 93 for *pfcrt* (76.2%), 81 for *pfmdr1* fragment 1 (66.4%), 70 for *pfmdr1* fragment 2 (57.4%), 86 for *pfdhps* fragment 1 (70.5%), 87 for *pfdhps* fragment 2 (71.3%) and 95 for *pfdhfr* (77.9%) (Sequencing success rate per gene and extraction method and per position can be found in Additional file 1: Tables S3 and S4, respectively). Unsuccessful sequencing was correlated with low parasite numbers (72.7% of these samples presented a density below 10 parasites/µl). No amplification was observed in the negative controls.

No significant difference was detected in resistance allele prevalence by extraction and collection methods, therefore these data were pooled together and allele prevalence was calculated for each gene and per locus (Table 3, Additional file 1: Table S5). Novel polymorphisms were detected in *pfmdr1* positions T1069T (derived from a T → G nucleotide change; in 4 out of 70 samples), T1071V (derived from an A → G nucleotide change; in 1 out of 70 samples) and S1075N (derived from a G → A nucleotide change; in 1 out of 70 samples) and in *k13* position S624L (derived from a C → T nucleotide change; in 1 out of 66 samples). An interesting side observation was that the control parasite line Dd2 for *pfmdr1* showed a mutation in position N86F, which derives from an A → T nucleotide change from N86Y, as previously reported by a different laboratory [24].

**Table 3:** Comparison of wild type and mutant frequencies of *P. falciparum* in mosquito samples using Sanger sequencing and NGS

Gene	Sanger sequencing of mosquito samples			NGS of mosquito samples^a^		Sequenced samples by both methods	p-value
	Wild type %	Mutant %	Mixture %	Wild type %	Mutant %		
*K13*							
Y493H R539T I543T C580Y	100	0	0	100	0	66	1
*pfcrt*							
CVMNK (72–76)	100	0	0	100	0	93	1
*pfmdr1* F1							
N86Y/F	98.8	0	1.2	100	0	81	3.44 × 10^−4^
Y184F	46.9	21.0	32.1	50.3	49.3		0.59
*pfmdr1* F2							
S1034C N1042D D1246Y	100	0	0	100	0	70	1
*pfdhps* F1							
S436F/A	100	0	0	100	0	86	1
A437G	2.3	95.3	2.3	0	100		1.32 × 10^−7^
*pfdhps* F2							
K540E	2.3	96.6	1.1	6.2	93.7	87	0.17
A581G A613T/S	100	0	0	100	0		1
*pfdhfr*							
C50R/S	99	1.0	0	100	0	95	1.64 × 10^−4^
N51I	0	100	0	0.9	98.4		1
C59R	3.2	32.6	64.2	3.6	96.3		1
S108N	0	100	0	0.3	96.3		1
164L	100	0	0	100	0		1

Fisher’s exact test was utilized for all comparisons except for *pfmdr1* position Y184F, for which χ-squared test was performed. Sanger sequencing mixtures were assumed as mutant for statistical calculations. F1: fragment 1. F2: fragment 2. Wild-type haplotypes are indicated on the left and mutant amino acids on the right of the position number (see Additional file 1: Tables S5 and S7)

^a^NGS allele frequency estimates were calculated by dividing individual unfiltered allele depth by total filtered depth, hence total values do not always add up to 100 (see “Methods” section) [61]

### Allele frequencies estimation using Next Generation Sequencing (NGS)

Deep sequencing returned between 2,273,242 and 112,206 reads depending on the fragment analysed. However, after SNP calling and filtering, confident number of reads was between 115,477 and 2,255,289 with a geometric mean of 299,566. False discovery rate (FDR) of NGS calculated using Benjamini–Hochberg procedure was below 1%. 3D7 control base calling returned as expected except for some point mutations in *pfdhps* (see Additional file 1: Table S6). However, conservative regions surrounding well-known polymorphisms remained unchanged, giving confidence in the SNPs observed from the mosquito samples. Estimated wild-type and mutant allele frequencies per gene and per position are represented in Table 3 and Additional file 1: Table S7.

### Comparison of Sanger and NGS methods

Most of allele frequencies obtained by NGS were similar if not identical to allele prevalence obtained by Sanger sequencing although, in general, a higher number of wild-type alleles were detected with NGS (Table 3). Nevertheless, three positions of three different genes had statistically significant different results when comparing both sequencing techniques: *pfmdr1* position N86Y/F, *pfdhps* position A437G and *pfdhfr* position C50R/S. These results were due to the appearance of one or two observations by Sanger while none by NGS (Additional file 1: Table S7). Visual inspection of the raw sequencing reads did reveal these mutations but the variant calling software filtered these out.

## Discussion

Here, using a Next Generation Sequencing platform, mutant allele frequencies were obtained of *P. falciparum* parasites isolated from mosquitoes from southern Mozambique. Similar allele frequencies of resistance markers were found with NGS compared to the prevalence of markers obtained with the gold standard Sanger sequencing. These resistance data obtained from mosquitoes involved a simpler and non-invasive sample collection, and the NGS approach allowed for high-throughput analyses leading to epidemiologically more relevant allele frequencies as opposed to resistance prevalence. Therefore, this mosquito-based NGS approach is a valuable drug resistance marker surveillance tool to fill in the large geographical gaps in resistance surveillance.

Both Sanger and NGS reflected 100% of prevalence of the wild-type allele of the *k13* propeller gene in positions Y493H, R539T, I543T and C580Y, polymorphisms associated with artemisinin resistance [36]. This finding along with other studies supports the notion that artemisinin-driven selection on the *k13* locus is still absent in Africa [23, 36]. However, Sanger sequencing revealed one not yet described polymorphism on the *k13* gene in one of the head/thorax samples in position S624L. Although the relevance of this single observation is uncertain, it has been shown that new point mutations frequently appear worldwide in the *k13* locus [36, 51–54] and, even though not being strongly selected at this time outside SE Asia, they have the potential to enable resistance to rapidly emerge in the future [13, 55]. Particularly with the recent observations of an independent emergence of the C580Y point mutation in Guyana [56] and the reporting of an artemisinin-resistant *P. falciparum* with a previously unreported SNP in position M579I contracted in Equatorial Guinea [54], *k13* molecular surveillance is of critical importance. With newly arising mutations starting at low frequencies in a population, allele frequency estimates, rather than prevalence estimates, are more reliable [36, 57]. This study further confirmed the decades long increase of wild-type *pfcrt* parasites in the area [23, 39], with no mutations at all observed on this locus compared to 85% presence of K76T mutation in 1999 [58]. As previously observed by Gupta and colleagues [23], Sanger sequencing analyses of *pfmdr1* revealed that more than half (53.1%) of the positive for *P. falciparum* mosquitoes tested exhibited Y184F mutation, including 32% of the total that accounts for mixed (wild-type and mutant) infections. NGS analyses were very similar with 49.3% frequency of this same point mutation. Furthermore, new possible point mutations appeared during Sanger sequencing analyses of *pfmdr1* in positions T1069T, T1071V and S1075N in five different mosquito abdomen samples. Positions T1071V and S1075N have not been previously described and were polymorphic in only one sample each. However, position T1069T has been previously reported [23] and showed the same mutation along 4 mosquito samples, which could maybe indicate a plausible novel mutation. On a different note, SP resistance linked to mutations in the *pfdhps* and *pfdhfr* genes in Africa is widespread [9]. In the study area, quintuple mutations of *pfdhps* and *pfdhfr* were nearly fixed. Yet, positions C50R/S and I164L of *pfdhfr* and S436F/A, A581G and A613T/S of *pfdhps* still remain wild-type according to results of both methods. Although it has been observed that *pfdhfr* polymorphism frequencies in mosquitoes may differ from those in humans [59], overall, our observations are within a similar range to those numbers obtained from human blood samples in other studies [60].

Sanger sequencing and NGS approaches gave overall very similar resistance markers estimates, in spite of some minor discrepancies. As previously mentioned, some of these discrepancies are due to the fact that Sanger will provide with prevalence approximations while NGS will measure allele frequency. While prevalence of resistance on a human subject level may inform treatment choice, on an epidemiological level the parameter of interest is allele frequency. Furthermore, frequency estimates also allow to capture minority emerging genotypes in mixed infections, which would be missed with prevalence analyses that do not detect mixed alleles below a threshold of 10–20% [3, 60, 61]. On the flipside, however, prevalence analysis would be more sensitive for the detection of mutants that are at a higher frequency within a given sample but at low frequency on a population level. The latter, however, would be less likely to occur for novel emerging mutations. The NGS approach allows for pooling of samples too, which reduces cost and performance time without compromise in information since the objective is population-level allele frequency [3]. Moreover, pooled deep sequencing offers high read coverage and sequencing depth and permits to increase sample size, as previously seen in other studies [3, 26, 27]. It also provides the means to monitor whole genes, which is necessary for those with multiple point mutations associated with resistance and which would allow the detection of novel SNPs usually not analysed with other detection techniques. Moreover, results from this study show that pooled deep sequencing of infected mosquito samples is a more suitable alternative to pooled human blood samples deep sequencing. It does not require specialized personnel to draw blood from patients and it avoids complex ethical requirements and visiting expeditions, which can sometimes be very challenging in low-income settings. Since data obtained from mosquitoes has been shown to correlate well with data from humans [23, 36, 39, 60], mosquitoes could be used as a sentinel group for resistance surveillance purposes.

A relatively high *P. falciparum* infection rate (23.6%) was observed in these mosquitoes collected in southern Mozambique, an area with high level of malaria transmission (between 100 and 200 cases per 1000 population) [29]. Of note however, was that extracts from head/thorax (6%) and abdomen (18.3%) were for unclear reasons significantly lower than isolates from whole mosquitoes. Further studies are also needed to confirm if only abdomens should be screened as according to our results they presented a higher positivity rate. However, it should be taken into account that there is the possibility to a change in allele proportions between head/thorax and abdomen portions. Nevertheless, as mentioned beforehand, for allele frequency purposes, however, a pooling strategy of whole mosquitoes would be adequate, such as previously demonstrated for the detection of dengue [62] and Ross River viruses [63], though with the limitation that any pooling strategy could bias results towards higher density infections. Interestingly, more *P. falciparum* positive mosquitoes appeared to be captured by miniature light traps than early morning collections using manual aspiration (Table 1). An intriguing hypothesis is that this could be due to behavioural manipulation: it is thought that *Plasmodium*-infected humans present an increased attractiveness to the arthropod vector [64] and that those mosquitoes infected with *P. falciparum* are more attracted to humans [65]. Moreover, it has explicitly been shown that *Aedes aegypti* infectious with *Plasmodium gallinaceum* present an increased host-seeking behaviour [66]. Because miniature light traps are located in close proximity to people sleeping under LLINs, this could explain the higher frequency of *P. falciparum* positive mosquitoes captured by the miniature light traps.

Although the proposed surveillance tool of NGS of *P. falciparum* isolates from mosquitoes is promising, there are some caveats. First, the validity of extrapolation of resistance marker frequency in mosquitoes to human population needs to be confirmed. Detection of resistance markers could be more sensitive in the human host—when de novo mutants could have been selected—instead of during the mosquito life cycle, when negative selection against mutants could occur. However, evidence for this effect is sparse (reviewed in [67]). Of note is that frequency of resistance surveillance in the mosquito vector is arguably a more relevant measure of resistance epidemiology as anti-malarial resistance is more likely to be transmitted than acquired (reviewed in [68]). Second, this study is based on a single mosquito species, *Anopheles funestus*, an indoor-biting highly antropophylic vector, and the extent to which different vector species carry different *P. falciparum* genotypes, and hence bias the allele frequency, is unknown. However, it has been observed that different *Plasmodium* genotypes are randomly distributed [69]. Third, these mosquitoes were collected in a relatively high transmission area. The approach may be less cost-effective in a low transmission area when a larger number of mosquitoes need to be screened and NGS will be less capable of detecting low-density infections. However, this is a general issue for resistance surveillance in low transmission areas, irrespective of using human or mosquito samples. Pooled screening approaches would significantly reduce this cost to more efficiently identify *P. falciparum* infected specimens with the caveat that pooling could bias results to high density mosquito samples. Fourth, as with many molecular approaches, this Illumina sequencing approach only allows the detection of known SNPs. Other approaches will be needed to detect novel mutations and gene duplications. Finally, NGS is a relatively new technique linked to uncertainty due to errors in alignment, base calling or filtering [50]. For example, unexpected mutations in positions S436F/A, A437G, A581G and A613T/S of *pfdhps* in the 3D7 control were found (see Additional file 1: Table S6) and statistically significant differences among Sanger and NGS results in *pfmdr1* position N86Y/F, *pfdhps* position A437G and *pfdhfr* position C50R/S. These statistically significant differences could possibly be overcome by moving to an individual NGS approach rather than a pooled one. However, they could still be either a false negative or positive by Sanger sequencing or by NGS or a distinction between prevalence and frequency. It is, therefore, imperative to account for uncertainty linked to NGS and to try to reduce it. There are different strategies to do so, although one must accept that there is not a perfect combination and that the fast development of bioinformatic tools means that recommendations may change very rapidly but also improve [50].

## Conclusions

In conclusion, pooled deep sequencing of *P. falciparum* isolates extracted from dried mosquitoes appears to be an efficient and cost-effective method to quantify allele frequencies at a population level. It is a promising technique, which allows not only to analyse known and unknown markers of resistance, but to detect them in mixed infections. Moreover, it allows to follow up rapid and radical population changes in a timelier manner as mosquitoes could be collected throughout the year and without a long planning phase of obtaining ethical approval. Therefore, this approach would allow active surveillance in an increasing number of sites in order to obtain information on molecular markers of resistance, which could be applied to current malaria control programmes.

## Supplementary information

**Additional file 1.** Additional figure and tables.

## Data Availability

The datasets used and/or analysed during the current study are available from the corresponding author on reasonable request.
