# Supplementary material for: Mosquitoes as a feasible sentinel group for anti-malarial resistance surveillance by Next Generation Sequencing of Plasmodium falciparum
Source: Malar J. 2019 Oct 17;18:351. doi: 10.1186/s12936-019-2946-0 (PMC6796398; doi:10.1186/s12936-019-2946-0)
Supplement: Supplementary file 1 — Additional file 1. Additional figure and tables. [file 12936_2019_2946_MOESM1_ESM.docx]

**Additional File 1 for**

**Mosquitoes as a feasible sentinel group for antimalarial resistance surveillance by Next Generation Sequencing of *Plasmodium falciparum***

Rebecca Smith-Aguasca, Himanshu Gupta, Estefanía Uberegui Bernad, Mara Maquinac, Francisco Saute, Krijn P. Paaijmans, Alfredo Mayor, Silvie Huijben.

To whom correspondence should be addressed: Dr. Silvie Huijben

E-mail: [shuijben@asu.edu](mailto:shuijben@asu.edu)

**This PDF file includes:**

**Fig. S1**

**Tables S1 to S7**

**References for SI reference citations**

**Additional Information Figure and Tables**


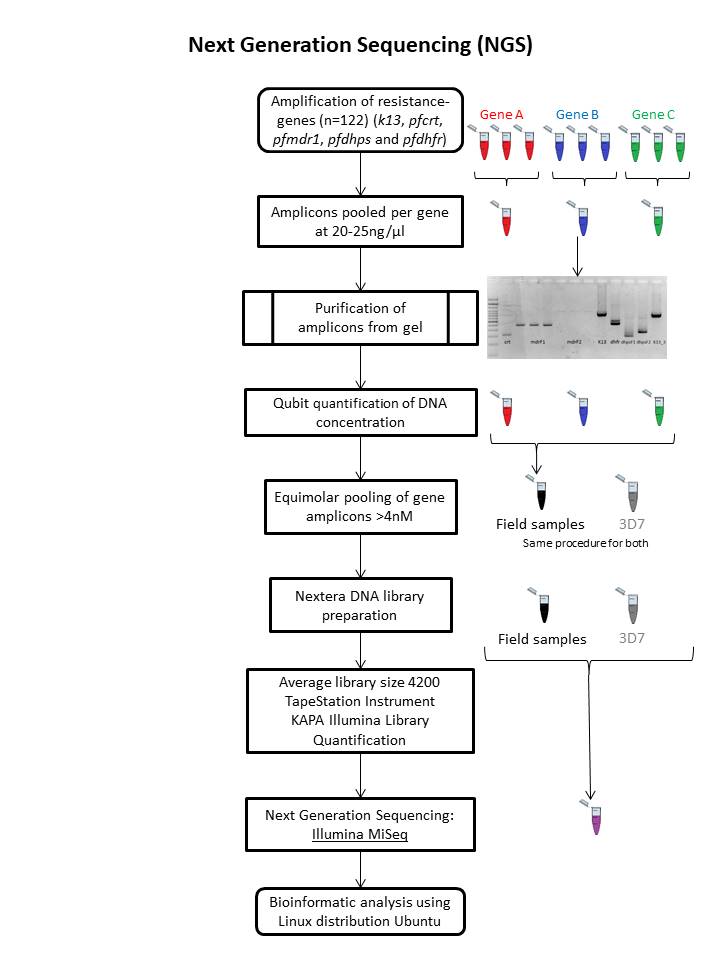


**Figure S1.** Next Generation Sequencing (NGS) schematic workflow.

**Table S1** Primers used for DNA amplification. *K13* propeller gene primers were taken from (1), *pfdhfr* from (2,3), and *pfdhps* from (2). *Pfcrt* primers were previously described in (4) and *pfmdr1* in (5). OuterF: outer reaction forward primer. OuterR: outer reaction reverse primer. NestedF: nested reaction forward primer. NestedR: nested reaction reverse primer. F1: fragment 1. F2: fragment 2.

| **Gene** | **Conventional PCR** | | | | |
| --- | --- | --- | --- | --- | --- |
|  | **PCR primers** | | **Annealing Tm (ºC)** | **Cycles** | **PCR size (bp)** |
| ***K13*** | **OuterF** | cggagtgaccaaatctggga | 58 | 30 | 2097 |
|  | **OuterR** | gggaatctggtggtaacagc |  |  |  |
|  | **NestedF** | gccaagctgccattcatttg | 60 | 40 | 849 |
|  | **NestedR** | gccttgttgaaagaagcaga |  |  |  |
| ***pfcrt*** | **OuterF** | tttaggtggaggttcttgtctt | 52 | 25 | 569 |
|  | **OuterR** | atacttaattgaagaacaaatgattgga |  |  |  |
|  | **NestedF** | tttaggtggaggttcttgtctt | 50 | 35 | 319 |
|  | **NestedR** | ttggtaggtggaatagattctct |  |  |  |
| ***pfmdr1*** | **F1 F** | agagaaaaaagatggtaacctcag | 60 | 35 | 610 |
|  | **F1 R** | accacaaacataaattaacgg |  |  |  |
|  | **F2 F** | accccaggtgttttatctgc | 60 | 35 | 1295 |
|  | **F2 R** | tgtccacctgataagcttttacc |  |  |  |
| ***pfdhps*** | **OuterF** | aacctaaacgtgctgttcaa | 56 | 30 | 711 |
|  | **OuterR** | aattgtgtgatttgtccacaa |  |  |  |
|  | **NestedF F1** | aacctaaacgtgctgttcaa | 56 | 30 | 306 |
|  | **NestedR F1** | ctggattatttgtacaagcac |  |  |  |
|  | **NestedF F2** | caaattctatagtgtagttc | 56 | 30 | 377 |
|  | **NestedR F2** | aattgtgtgatttgtccacaa |  |  |  |
| ***pfdhfr*** | **OuterF** | tcctttttatgatggaacaag | 56 | 30 | 653 |
|  | **OuterR** | agtatatacatcgctaacaga |  |  |  |
|  | **NestedF** | tgaggtttttaataactacacatttagaggtct | 51 | 30 | 549 |
|  | **NestedR** | tcgctaacagaaataatttgatactcat |  |  |  |

**Table S2.** Primers used for DNA sequencing. *K13* propeller gene primers were taken from (1), *pfdhfr* from (3), and *pfdhps* from (2). *Pfcrt* primers were previously described in (4) and *pfmdr1* in (5). F1: fragment 1. F2: fragment 2.

| **Gene** | **Sequencing primers** | | **Sequencing size (bp)** | **Covered aminoacids** |
| --- | --- | --- | --- | --- |
| ***K13*** | **Forward** | gccaagctgccattcatttg | 849 | 427-709 |
|  | **Reverse** | gccttgttgaaagaagcaga |  |  |
| ***pfcrt*** | **Forward** | tttaggtggaggttcttgtctt | 319 | 35 - 120 |
|  | **Reverse** | ttggtaggtggaatagattctct |  |  |
| ***pfmdr1*** | **F1 Forward** | ccgtttaaatgtttacctgcac | 495 | 45 – 209 |
|  | **F1 Reverse** | accacaaacataaattaacgg |  |  |
|  | **F2 Forward** | tttttaatacaggaagcatttt | 884 | 984 – 1277 |
|  | **F2 Reverse** | aatgttgcatcttctcttc |  |  |
| ***pfdhps*** | **F1 Forward** | aacctaaacgtgctgttcaa | 306 | 411 - 511 |
|  | **F1 Reverse** | ctggattatttgtacaagcac |  |  |
|  | **F2 Forward** | caaattctatagtgtagttc | 377 | 523 - 646 |
|  | **F2 Reverse** | aattgtgtgatttgtccacaa |  |  |
| ***pfdhfr*** | **Forward** | tgaggtttttaataactacacatttagaggtct | 549 | 30 - 210 |
|  | **Reverse** | tcgctaacagaaataatttgatactcat |  |  |

**Table S3.** Successful sequenced samples (both or only one forward or reverse) per extraction method. F1: fragment 1. F2: fragment 2. Pooled: 5 mosquitoes.

| **Gene** | | **Whole body** | **Head/thorax** | **Abdomen** | **Pooled** | **Total** |
| --- | --- | --- | --- | --- | --- | --- |
| ***K13*** | | 20/34 | 5/18 | 28/57 | 13/13 | 66/122 |
| ***pfcrt*** | | 24/34 | 12/18 | 44/57 | 13/13 | 93/122 |
| ***pfmdr1*** | **F1** | 26/34 | 8/18 | 34/57 | 13/13 | 81/122 |
|  | **F2** | 20/34 | 6/18 | 33/57 | 11/13 | 70/122 |
| ***pfdhps*** | **F1** | 23/34 | 9/18 | 41/57 | 13/13 | 86/122 |
|  | **F2** | 24/34 | 9/18 | 41/57 | 13/13 | 87/122 |
| ***pfdhfr*** | | 26/34 | 11/18 | 45/57 | 13/13 | 95/122 |

**Table S4.** Success rate (%) per position of sequenced samples; out of those samples which had both or only one forward or reverse sequenced. F: forward. R: reverse. Wild-type haplotypes are indicated on the left and mutant amino acids on the right of the position number.

| **Gene** | | **Position** | **F+R success (%)** | **F or R success (%)** |
| --- | --- | --- | --- | --- |
| ***K13*** | | **Y493H** | 65/66 (98.5) | 1/66 (1.5) |
|  |  | **R539T** | 65/66 (98.5) | 1/66 (1.5) |
|  |  | **I543T** | 65/66 (98.5) | 1/66 (1.5) |
|  |  | **C580Y** | 65/66 (98.5) | 1/66 (1.5) |
| ***pfcrt*** | | **72 – 76 CVMNK** | 93/93 (100) | 0/93 (0) |
| ***pfmdr1*** | **F1** | **N86Y** | 71/81 (87.7) | 10/81 (12.3) |
|  |  | **Y184F** | 65/81 (80.2) | 16/81 (19.8) |
|  | **F2** | **S1034C, N1042D, D1246Y** | 210/210 (100) | 0/210 (0) |
| ***pfdhps*** | **F1** | **S436A/F** | 85/86 (98.8) | 1/86 (1.2) |
|  |  | **A437G** | 85/86 (98.8) | 1/86 (1.2) |
|  | **F2** | **K540E** | 42/87 (48.3) | 45/87 (51.7) |
|  |  | **A581G** | 86/87 (98.9) | 1/87 (1.1) |
|  |  | **A613T/S** | 86/87 (98.9) | 1/87 (1.1) |
| ***pfdhfr*** | | **C50R** | 82/95 (86.3) | 13/95 (13.7) |
|  |  | **N51I** | 82/95 (86.3) | 13/95 (13.7) |
|  |  | **C59R** | 86/95 (90.5) | 9/95 (9.5) |
|  |  | **S108N** | 95/95 (100) | 0/95 (0) |
|  |  | **I164L** | 95/95 (100) | 0/95 (0) |

**Table S5.** Resistance allele frequency (%) per gene and position by Sanger Sequencing. F1: fragment 1. F2: fragment 2. n: numbers positive. N: numbers tested. Wild-type haplotypes are indicated on the left and mutant amino acids on the right of the position number.

| **Gene** | | **Wild type n/N (%)** | **Mutant n/N (%)** | **Mixed n/N (%)** |
| --- | --- | --- | --- | --- |
| ***K13*** | **Y493H R539T I543T C580Y** | 66/66 (100) | 0/66 (0) | 0/66 (0) |
| ***pfcrt*** | **CVMNK** | 93/93 (100) | 0/93 (0) | 0/93 (0) |
| ***pfmdr1 F1*** | **N86Y/F** | 80/81 (98.8) | 0/81 (0) | 1/81 (1.2) |
|  | **Y184F** | 38/81 (46.9) | 17/81 (21.0) | 26/81 (32.1) |
| ***pfmdr1 F2*** | **S1034C N1042D D1246Y** | 70/70 (100) | 0/70 (0) | 0/70 (0) |
| ***pfdhps F1*** | **S436F/A** | 86/86 (100) | 0/86 (0) | 0/86 (0) |
|  | **A437G** | 2/86 (2.3) | 82/86 (95.3) | 2/86 (2.3) |
| ***pfdhps F2*** | **K540E** | 2/87 (2.3) | 84/87 (96.6) | 1/87 (1.1) |
|  | **A581G A613T/S** | 87/87 (100) | 0/87 (0) | 0/87 (0) |
| ***pfdhfr*** | **C50R/S** | 94/95 (99.0) | 1/95 (1.0) | 0/95 (0) |
|  | **N51I** | 0/95 (0) | 95/95 (100) | 0/95 (0) |
|  | **C59R** | 3/95 (3.2) | 31/95 (32.6) | 61/95 (64.2) |
|  | **S108N** | 0/95 (0) | 95/95 (100) | 0/95 (0) |
|  | **I164L** | 95/95 (100) | 0/95 (0) | 0/95 (0) |

**Table S6.** 3D7 profile for *pfdhps* observed with NGS analysis.

| **Position** | **Wild type (%)** | **Mutant (%)** |
| --- | --- | --- |
| **S436F/A** | **53.7** | **44.5** |
| **A437G** | **8.9** | **91.1** |
| **K540E** | **100** | **0** |
| **A581G** | **68.1** | **30.8** |
| **A613T/S** | **65.7** | **31** |

**Table S7.** Allele frequency (%) per gene and position by NGS. F1: fragment 1. F2: fragment 2. n: numbers positive. N: numbers tested. Wild-type haplotypes are indicated on the left and mutant amino acids on the right of the position number. Allele frequency estimates were calculated by dividing individual unfiltered allele depth by total filtered depth, hence total values do not always add up to 100 (See Methods section) (6).

| **Gene** | | **Replicate 1** | | **Replicate 2** | | **Combined** | |
| --- | --- | --- | --- | --- | --- | --- | --- |
|  |  | **Wild type (%)** | **Mutant (%)** | **Wild type (%)** | **Mutant (%)** | **Wild type n/N (%)** | **Mutant n/N (%)** |
| ***K13*** | **Y493H R539T I543T C580Y** | 100 | 0 | 100 | 0 | 2,255,289/2,255,289 (100) | 0/2,255,289 (0) |
| ***pfcrt*** | **CVMNK (72-76)** | 100 | 0 | 100 | 0 | 162,370/162,370 (100) | 0/162,370 (0) |
| ***pfmdr1* F1** | **N86Y/F** | 100 | 0 | 100 | 0 | 235,479/235,479 (100) | 0/235,479 (0) |
|  | **Y184F** | 50.3 | 49.2 | 50.2 | 49.4 | 174,438/434,154 (50.3) | 170,979/434,154 (49.3) |
| ***pfmdr1* F2** | **S1034C N1042D D1246Y** | 100 | 0 | 100 | 0 | 803,979/803,979 (100) | 0/803,979 (0) |
| ***pfdhps* F1** | **S436F/A** | 100 | 0 | 100 | 0 | 235,479/235,479 (100) | 0/235,479 (0) |
|  | **A437G** | 0 | 100 | 0 | 100 | 0/235,479 (0) | 235,479/235,479 (100) |
| ***pfdhps* F2** | **K540E** | 6.1 | 93.7 | 6.2 | 93.6 | 10,796/175,620 (6.2) | 164,599/175,620 (93.7) |
|  | **A581G A613T/S** | 100 | 0 | 100 | 0 | 308,862/308,862 (100) | 0/308,862 (0) |
| ***pfdhfr*** | **C50R/S** | 100 | 0 | 100 | 0 | 578,296/578,296 (100) | 0/578,296 (0) |
|  | **N51I** | 0.9 | 98.3 | 0.8 | 98.4 | 2,404/278,704 (0.9) | 274,121/278,704 (98.4) |
|  | **C59R** | 3.5 | 96.3 | 3.6 | 96.2 | 11,636/327,444 (3.6) | 315,252/327,444 (96.3) |
|  | **S108N** | 0.3 | 99.5 | 0.3 | 99.5 | 1,917/562,631 (0.3) | 559,631/562,631 (99.5) |
|  | **I164L** | 100 | 0 | 100 | 0 | 578,296/578,296 (100) | 0/578,296 (0) |

**References**

1. Ménard D, Khim N, Beghain J, Adegnika AA, Shafiul-Alam M, Amodu O, et al. A Worldwide Map of Plasmodium falciparum *K13*-Propeller Polymorphisms. N Engl J Med [Internet]. 2016 Jun 23 [cited 2017 May 28];374(25):2453–64. Available from: http://www.nejm.org/doi/10.1056/NEJMoa1513137

2. Taylor SM, Parobek CM, Aragam N, Ngasala BE, Martensson A, Meshnick SR, et al. Pooled Deep Sequencing of *Plasmodium falciparum* Isolates: An Efficient and Scalable Tool to Quantify Prevailing Malaria Drug-Resistance Genotypes. J Infect Dis [Internet]. 2013 Dec 15 [cited 2017 Jan 10];208(12):1998–2006. Available from: http://www.ncbi.nlm.nih.gov/pubmed/23908494

3. Taylor SM, Antonia A, Feng G, Mwapasa V, Chaluluka E, Molyneux M, et al. Adaptive evolution and fixation of drug-resistant *Plasmodium falciparum* genotypes in pregnancy-associated malaria: 9-year results from the QuEERPAM study. Infect Genet Evol [Internet]. 2012 Mar [cited 2017 May 30];12(2):282–90. Available from: http://www.ncbi.nlm.nih.gov/pubmed/22119749

4. Galatas B, Nhamussua L, Candrinho B, Mabote L, Cisteró P, Gupta H, et al. In-Vivo Efficacy of Chloroquine to Clear Asymptomatic Infections in Mozambican Adults: A Randomized, Placebo-controlled Trial with Implications for Elimination Strategies. Sci Rep [Internet]. 2017 Dec 2 [cited 2017 Jun 6];7(1):1356. Available from: http://www.ncbi.nlm.nih.gov/pubmed/28465550

5. Gupta H, Macete E, Bulo H, Salvador C, Warsame M, Carvalho E, et al. Drug-Resistant Polymorphisms and Copy Numbers in Plasmodium falciparum, Mozambique, 2015. Emerg Infect Dis [Internet]. 2018 Jan [cited 2018 Nov 2];24(1):40–8. Available from: http://www.ncbi.nlm.nih.gov/pubmed/29260689

6. Van der Auwera GA, Carneiro MO, Hartl C, Poplin R, Del Angel G, Levy-Moonshine A, et al. From FastQ data to high confidence variant calls: the Genome Analysis Toolkit best practices pipeline. Curr Protoc Bioinforma [Internet]. 2013 [cited 2018 Feb 20];43(1110):11.10.1-33. Available from: http://www.ncbi.nlm.nih.gov/pubmed/25431634
